# The impact of diet and lifestyle on wellbeing in adults during COVID-19 lockdown

**DOI:** 10.3389/fnut.2022.993180

**Published:** 2022-10-06

**Authors:** Anne-Katrin Muth, Annabel Losecaat Vermeer, Damiano Terenzi, Soyoung Q. Park

**Affiliations:** ^1^Department of Decision Neuroscience and Nutrition, German Institute of Human Nutrition (DIfE), Potsdam-Rehbrücke, Germany; ^2^Neuroscience Research Center, and Berlin Institute of Health, Charité-Universitätsmedizin Berlin, Corporate Member of Freie Universität Berlin, Humboldt-Universität zu Berlin, Berlin, Germany; ^3^Deutsches Zentrum für Diabetes, Neuherberg, Germany

**Keywords:** eating behavior, mental health, COVID-19, gender, activity

## Abstract

A healthy diet and lifestyle may protect against adverse mental health outcomes, which is especially crucial during stressful times, such as the COVID-19 pandemic. This preregistered longitudinal online study explored whether diet and lifestyle (physical activity, sleep, and social interactions) were associated with wellbeing and mood during a light lockdown in Germany. Participants (*N* = 117, 72 males; 28 ± 9 years old) answered mental health and lifestyle questionnaires (social connections, sleep, activity) followed by submitting 1 week of food and mood-lifestyle diary (food intake, positive and negative mood, mental wellbeing, sleep quality, physical activity level, quantity and quality of social interactions) *via* a smartphone app. We used multivariate linear and mixed-effects models to associate mood and wellbeing with dietary components and lifestyle factors. Interindividual analyses revealed that sleep and social interaction significantly impacted mood and wellbeing. Interestingly, fruit and vegetable intake correlated with wellbeing, even when controlling for all lifestyle factors. Fruit and vegetable intake also significantly correlated with daily fluctuations in wellbeing within individuals next to sleep, physical activity, and social interactions. We observed gender differences in fruit and vegetable intake and anxiety levels. Our results emphasize the importance of diet contributing to individual wellbeing, even in the challenging times of a pandemic. Future research is necessary to test if our findings could extend to other populations.

## Introduction

COVID-19 lockdowns and social isolation have taken a toll on mental wellbeing (1–3). Lifestyle factors, including diet and physical activity, are shown to effectively reduce the risk of mental health disorders (4). However, it is unclear whether and how such lifestyle factors contribute to mental wellbeing during the pandemic.

A diet high in fruit and vegetables reduced depression risk (5–7) and anxiety (8). On the other hand, diets high in trans fatty acids from processed foods (9) and fast food increased depression risk over 6-years (10, 11). Dietary intake can have relatively instant effects on mood and wellbeing. Studies investigating daily associations found that higher fruit and vegetable intake was associated with wellbeing (12) and positive mood the same day or the next day (13). While eating salty snacks correlated with higher negative mood the next day in people with a high Body Mass Index (BMI) (13). Similarly, higher saturated fat intake correlated with negative mood 2 days later in college students (14).

Importantly, diet-induced neuroinflammation is a key mechanism linking diet, cognitive function, and even gray matter volume loss (15). The dietary inflammatory index (DII) estimates a diet’s inflammatory potential (16), and at least two meta-analyses have established a link with depression (17, 18), depressive symptoms, anxiety, and psychological distress (19, 20). Importantly, DII and mental health profiles were less associated in men than in women (19), pointing to gender differences.

Besides diet, physical activity and sleep play a major role in wellbeing (21, 22), depression (23), anxiety (24, 25) and sleep quality (26). However, the pandemic has impacted lifestyle behaviors. For example, a recent study demonstrated that roughly 53% of 5,000 participants reported a change in activity level during the COVID-19 pandemic (27). Sleep disturbances were reliably associated with the risk for depressive symptoms and clinical depression (4) and correlated positively with mental health issues (28), suggesting that physical activity and sleep quality majorly contribute to wellbeing and mood during the pandemic.

Managing the COVID-19 pandemic required social distancing, making the link between social interaction and mental health outcomes of high interest. Social interaction is vital for mental health outcomes, including wellbeing and symptoms of depression or anxiety (29–31). For example, loneliness, the subjective feeling of the absence of a social network or a companion, is associated with adverse physical and mental health outcomes (30) and low physical activity levels in mental health patient groups (32, 33). During COVID-19-lockdown, social distancing restrictions led to increased feelings of social isolation, which coincided with more severe mental health outcomes (34). At the same time, a good relationship quality was crucial in maintaining mental health (3). Furthermore, wellbeing during the pandemic was associated with satisfaction of psychological needs at an inter- and intrapersonal level (35). Data from an Italian study during lockdown and when some restrictions were lifted showed that both emotional eating and binge-eating were increased in the presence of emotional distress, including higher levels of anxiety and depression, but also partially correlated with relationship quality and quality of life (36). An interesting question that remains is to what extent dietary intake can ameliorate the negative consequences of living through a pandemic in the context of physical activity, sleep, and social interaction quality.

In this preregistered online study,^1^ we investigated whether diet, lifestyle factors, and social interaction were associated with wellbeing, anxiety, and feeling of excitement during COVID-19 lockdown. We hypothesized that food intake (i.e., fat, carbohydrates, fruit and vegetables) contributes significantly to (1) individual wellbeing, (2) anxiety, and (3) excitement, even when controlling for lifestyle factors. Next to these preregistered analyses, we tested whether inflammation, as a possible mechanism, plays a role in the relationship between food intake and wellbeing.

## Materials and methods

### Participants

We recruited participants *via* the online research platform Prolific. German-speaking individuals without prior mental health diagnoses, residing in Germany at the time of the study, with an Apple or Android smartphone for using the FoodApp, were eligible to participate. We excluded participants who showed above-threshold depressive symptoms (i.e., above 30, which is classified as “severe”) determined by the Beck Depression Inventory [BDI; German version (37)]. Questionnaires were completed online on the SoSci Survey platform. The food and mood diary records were recorded using the FoodApp available for Android and Apple smartphones. Participants provided informed consent and received £28 for participation. Ethical approval was obtained from the Humboldt University of Berlin.

### Study design

**FIGURE 1:**
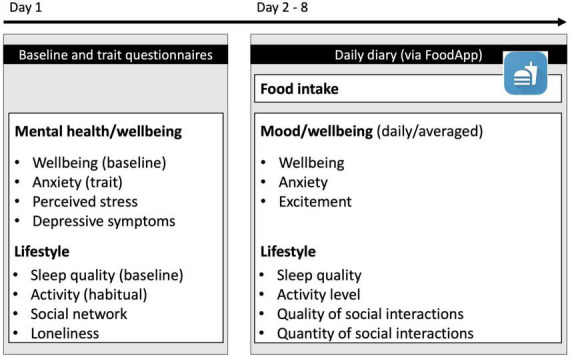
Study outline. Participants first answered questionnaires assessing baseline mental health and wellbeing as well as lifestyle-related questionnaires. Next, they completed a daily food, mood, and lifestyle diary for 7 days *via* a smartphone app.

We conducted an online study using questionnaires assessing mental health, wellbeing, and lifestyle factors. Afterward, participants kept a food and mood diary and a record of sleep quality, activity, and social interactions for 7 days (Figure 1). In particular, in this study we wanted to investigate the relationship between food intake as independent variables (i.e., fruit and vegetable, fat and carbohydrate intake) and mood (i.e., wellbeing, anxiety, excitement) as dependent variables while controlling for lifestyle factors (i.e., activity, sleep and social interaction quality and quantity). Data were collected between 11 and 24 November 2020 at which time there was a light lockdown in Germany. During this time, people were asked to reduce social contacts to the minimum. In public, one was only allowed to meet with people of one’s household and one additional household (from: 28.10.2020).^2^

### Assessment of food-mood and lifestyle diary

The food-mood and lifestyle diaries were completed using a smartphone FoodApp for 7 days [following (14)]. For food intake, we recorded the following information: date, time, type of meal, companionship during the meal, food items, and weight consumed. Food items could be chosen from a list of about 10,000 food and beverage items commonly available in Germany, for example, “potatoes peeled boiled” or “wholemeal bread with margarine and currant jam.” Participants chose the food item matching their consumption along with an estimate of how much they consumed in grams or milliliters. Participants were free to log their food intake after a meal, or later during the day. A reminder was sent to participants who did not submit their data by 7 p.m. that day. Dietary intake was evaluated using the German Federal Food Key data table [Bundeslebensmittelschlüssel (38)] made available by the Max-Rubner Institut (MRI). Data from days with extreme daily caloric intake were excluded from analysis (for women: < 500 or > 3,500 kcal/day, for men: < 800 and > 4,000 kcal/day considered as unrealistic amounts) following (39).

For dietary intake, we calculated energy-adjusted (ea) values to account for an individual’s total energy intake (i.e., g/1,000 kcal/day) as suggested by Agnoli et al. (40). Additionally, we computed daily energy derived from each macronutrient. For this, we multiplied the daily intake of carbohydrate and protein (g/d) by 4 kcal, and fat intake by 9 kcal (Table 1). Outliers in dietary data were winsorized separately for men and women.

**TABLE 1:** Sample characteristics by gender.

	Total (*N* = 117)^a^	Women (*N* = 45)^a^	Men (*N* = 72)^a^	*p*-value^b^
Age	28.12 (8.91)	30.76 (10.44)	26.47 (7.42)	**0.009**
BMI	24.21 (4.18)	23.50 (4.27)	24.65 (4.09)	**0.016**
**Daily averaged food intake**				
Kilocalories	1,727.09 (504.04)	1,513.31 (437.25)	1,860.71 (499.53)	**<0.001**
Protein% of kcal	16.44 (4.26)	15.27 (2.62)	17.17 (4.89)	**0.020**
Carbohydrate% of kcal	47.85 (6.79)	48.49 (7.20)	47.44 (6.54)	0.4
Fat% of kcal	34.47 (6.59)	34.81 (7.90)	34.25 (5.67)	0.8
Fruit and vegetable (g/1,000 kcal)	73.82 (43.70)	91.96 (35.17)	62.48 (44.89)	**0.009**
Dietary inflammatory score	0.00 (1.92)	0.15 (1.86)	–0.09 (1.97)	0.6
**Daily averaged mood and lifestyle factors**				
Wellbeing	22.26 (2.84)	22.11 (3.01)	22.35 (2.75)	>0.9
Excitement	3.07 (0.64)	3.01 (0.74)	3.10 (0.56)	0.6
Anxiety	1.88 (0.66)	2.10 (0.65)	1.74 (0.64)	**0.003**
Sleep quality	60.89 (17.42)	57.75 (17.26)	62.86 (17.36)	0.2
Activity level	41.21 (18.82)	43.09 (16.31)	40.04 (20.25)	0.3
SI^c^ quality	64.22 (14.26)	67.56 (14.92)	62.14 (13.52)	**0.021**
SI^c^ quantity	52.79 (19.10)	56.62 (18.36)	50.39 (19.29)	0.14
**Baseline and trait questionnaires**				
Baseline wellbeing	46.35 (9.05)	45.42 (9.33)	46.93 (8.89)	0.5
Trait anxiety	41.38 (12.15)	44.51 (12.54)	39.42 (11.57)	**0.035**
Depressive symptoms	9.26 (6.19)	10.13 (6.77)	8.71 (5.77)	0.4
Perceived stress	43.85 (18.18)	47.81 (18.81)	41.37 (17.45)	0.12

^a^Mean (SD); *n* (%).

^b^Wilcoxon rank-sum test; Pearson’s Chi-squared test; Fisher’s exact test.

^c^Social interaction. The bold values mean *p* < 0.05.

Finally, we calculated the Dietary Inflammatory Index (DII) score for each participant following (16). First, we selected the nutrients available to us, then we calculated z-scores by subtracting the standard global mean and dividing by the global standard deviation (the standard global mean and deviation are both found in Table 2 of Shivappa et al. (16). Then, we converted these z-scores to normal percentiles and multiplied them by 2, and subtracted them by 1. Each score was multiplied by its respective inflammatory effect score. Lastly, all scores were summed up to derive the overall DII score for each participant.

**TABLE 2:** Association between diet and lifestyle factors and measures of wellbeing and mood, using multiple linear regression models.

DV	IV	Coefficient	95% CI	*P*
Wellbeing	Intercept	6.48	–1.10–14.07	0.093
	Fruit and vegetable	0.01	0.00–0.02	**0.013**
	Fat	0.01	–0.06–0.08	0.725
	Carbohydrates	0.03	–0.01–0.08	0.142
	Activity	0.02	–0.00–0.04	0.067
	Sleep	0.05	0.03–0.07	**<0.001**
	SI^a^ quality	0.10	0.06–0.13	**<0.001**
	SI^a^ quantity	0.00	–0.02–0.02	0.996
	Gender (male)	0.86	0.01–1.71	**0.048**
	
	R^2^/R^2^ adjusted	0.528/0.493		

Anxiety	Intercept	1.91	–0.42–4.24	0.107
	Fruit and vegetable	–0.00	–0.01–0.00	0.171
	Fat	0.01	–0.01–0.03	0.289
	Carbohydrates	0.01	–0.01–0.02	0.396
	Activity	0.00	–0.01–0.01	0.776
	Sleep	–0.00	–0.01–0.00	0.452
	SI^a^ quality	–0.01	–0.02 to -0.00	**0.007**
	SI^a^ quantity	0.01	–0.00–0.01	0.145
	Gender (male)	–0.45	–0.72 to -0.19	**0.001**
	
	R^2^/R^2^ adjusted	0.186/0.125		

Excitement	Intercept	1.52	–0.63–3.68	0.164
	Fruit and vegetable	–0.00	–0.00–0.00	0.999
	Fat	–0.00	–0.02–0.02	0.707
	Carbohydrates	0.00	–0.01–0.01	0.956
	Activity	0.01	–0.00–0.01	0.111
	Sleep	0.01	0.00–0.02	**0.012**
	SI^a^ quality	0.01	–0.00–0.02	0.062
	SI^a^ quantity	0.01	–0.00–0.01	0.096
	Gender (male)	0.14	–0.10–0.38	0.252
	
	*R*^2^/*R*^2^ adjusted	0.242/0.186		

^a^Social interaction. All independent variables were entered simultaneously. The bold values mean *p* < 0.05.

Mood and lifestyle ratings were unlocked after 5 p.m. each day. Participants rated their wellbeing [using the short Warwick-Edinburgh Mental Wellbeing Scale (41)], anxiety, and excitement levels on a 5-point Likert scale. We added excitement and anxiety to daily measures to supplement functional wellbeing. Finally, sleep quality, activity level, quantity, and quality of social interactions were rated on a scale from 1 to 100.

### Questionnaires

We used the Warwick Edinburgh Mental Wellbeing Scale [WEMWBS (41)] to assess baseline wellbeing. This 14-item questionnaire assesses different aspects of positive mental health including balance of feeling and functioning. Example items include, “I’ve been feeling optimistic about the future” and “I’ve been thinking clearly.” We used the 7-item short form of the WEMWBS to assess daily wellbeing during the week of food-mood-lifestyle diary entries. This scale emphasizes functioning items over feeling items. Both versions are responsive to change (42).

Participants also completed mental health and lifestyle questionnaires, including trait anxiety [STAI (43)], depressive symptoms [BDI; German version (37)], and perceived stress [PSQ (44)]. Finally, the Community Assessment of Psychic Experiences (45) was analyzed as part of a separate study.

### Statistical analyses

All data was downloaded from the FoodApp server, Prolific, and SoSci survey and imported into R studio. Plots were made using ggstatsplot (46). We reported descriptive statistics for demographic characteristics, food intake, daily ratings as well as baseline and trait questionnaire scores.

### Weekly averages of daily data

First, we examined between-person relationships with each averaged daily dependent variable (wellbeing, anxiety, and excitement) separately. Independent variables were fruit and vegetable, fat and carbohydrate intake and lifestyle behaviors (i.e., activity, sleep, social interaction). We performed multiple linear regression using the *stats* package (47). The full models were specified as shown in equation (1). Gender was dummy-coded.


(1)⁢DV∼fruit&vegetables+fat+carbohydrate

+activity+sleep+quality⁢of⁢social⁢interaction

+quantity⁢of⁢social⁢interaction+gender

### Mediation analyses

To investigate if averaged daily measures of lifestyle mediated an effect of fruit and vegetable intake on wellbeing, we performed simple mediation analyses using the *MeMoBootR* package (48). We wanted to conduct three separate mediation analyses for the outcome variable wellbeing. The mediator variables were averaged from the daily diary; (1) physical activity, (2) sleep, and (3) social behavior. Covariates were, fat, carbohydrate, sleep, quality and quantity of social interaction, and gender.

### Daily and lagged analyses

Next, we performed same-day and 1- and 2-day lagged analyses to test intra-individual relationships between dependent variables (daily wellbeing, anxiety, excitement) and independent variables (i.e., fruit and vegetable, fat and carbohydrate intake) using multilevel modeling using the lme4 package (49). We included fruit and vegetable, fat and carbohydrate each as the level-1 independent variables and daily wellbeing, anxiety, excitement each as the level-1 outcome. We also included the dependent variable’s score of the previous day as a covariate (DV_T0_).

We assessed same-day associations between fruit and vegetable, fat and carbohydrate intake, wellbeing, anxiety and excitement along with lifestyle covariates [T1; see equation (2)].

One-day lagged associations tested whether eating fruit and vegetable, fat or carbohydrate intake on 1 day (T0) correlated with changes in wellbeing, anxiety and excitement the next day (T1) while controlling for mood on the first day. Lifestyle variables (i.e., activity, sleep, social interactions) were entered as covariates and not lagged [see Equation (3)].

Similarly, 2-day lagged analyses tested whether eating fruit and vegetables, carbohydrates, or dietary fats on 1 day (T0) were associated with wellbeing, anxiety or excitement 2 days later [T2; see Equation (4)]. Gender was dummy-coded.


(2)⁢DVT⁢1⁢∼fruit&vegetables⁢T1⁢+fat⁢T1⁢

+carbohydrate⁢T1⁢+activity⁢T1⁢+sleep⁢T1⁢

+quality⁢of⁢social⁢interaction⁢T1⁢+quantity⁢of⁢social

interaction⁢T1⁢+gender+DVT0⁢+⁢(1⁢|id)

(3)⁢DVT⁢1⁢∼fruit&vegetables⁢T0⁢+fatT0⁢

+carbohydrateT0⁢+activity⁢T1⁢+sleep⁢T1⁢+quality

of⁢social⁢interaction⁢T1⁢+quantity⁢of⁢social

interaction⁢T1⁢+gender+DVT0⁢+⁢(1⁢|id)

(4)⁢DVT⁢2⁢∼fruit&vegetables⁢T0⁢+fatT0⁢

+carbohydrateT0⁢+activity⁢T2⁢+sleep⁢T2⁢+quality⁢of

social⁢interaction⁢T2⁢+quantity⁢of⁢social⁢interaction⁢T2⁢

+gender+DVT1⁢+⁢(1⁢|id)

### Exploratory analyses

Exploratory associations between self-reported average fruit and vegetable, fat and carbohydrate intake, sleep, activity, social interaction quality and quantity and mental health questionnaires were tested with Pearson correlations. Significance levels were Bonferroni-corrected for multiple comparisons for each DV separately. Estimated marginal means analysis allowed us to test independent variable × gender effects on wellbeing and were carried out using the *emmeans* package (50). Mediation with covariates was conducted using the *MeMoBootR* package (48).

### Preregistration

Preregistered hypotheses and analyses are available on the public data repository Open Science Framework (see text footnote 1). We had not preregistered analysis by gender initially, however, after a more in-depth literature analysis it became clear, that gender differences play a larger role than we had previously assumed (8, 19). Therefore, we included gender as a covariate in all models, and tested correlations between wellbeing and (a) fruit and vegetable intake; and (b) social interaction quality stratified by gender.

We intended to include baseline wellbeing as a covariate in the wellbeing model, and similarly, perceived stress (PSQ) and trait anxiety (STAI) as covariates in the anxiety weekly averaged models. However, after observing high correlation between these measures we decided not to include these to avoid biased coefficients (51). In the mixed-effects models we included their wellbeing, anxiety, or excitement levels of the previous day as a covariate following (13) to test associations with daily wellbeing, anxiety, and excitement.

Finally, we originally wanted to use difference scores between habitual and concurrent lifestyle behaviors as mediators. However, at the time of conducting the study, light lockdown had been re-instated for more than 2 weeks. We reasoned that habitual data would reflect lockdown habits rather than pre-lockdown behaviors. Therefore, we used concurrent data of lifestyle behaviors instead.

## Results

### Participants

A total of 135 individuals participated in the study. After data collection, we excluded participants with severe symptom severity on the BDI (> 30, *N* = 3) as well as participants who logged fewer than 4 days of food intake and mood diary (*N* = 15). This resulted in a total sample of 117 participants (women *N* = 45, men *N* = 72, other = 0). Prior to the study, a power analysis based on a small effect size (f = 0.15), alpha = 0.05, and power of 0.95, estimated a required sample size of 119. Our final sample of *N* = 117 would deem sufficient.

**FIGURE 2:**
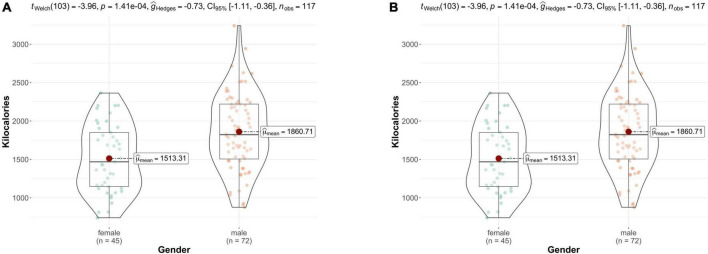
**(A)** Intake of kilocalories by gender; **(B)** energy adjusted fruit and vegetable intake by gender. Female participants consumed significantly more fruit and vegetables adjusted for total energy intake. Bars represent the interquartile range, with the median drawn in the middle. Whiskers depict the minimum and maximum values.

Averages of daily mood ratings and lifestyle factors are reported alongside baseline and trait questionnaire scores in Table 1. As shown in this table, in our sample women were significantly older than men, and had a lower BMI on average. Intake of kilocalories also differed between men and women (Figure 2A), whereby men had a higher total energy intake and consumed more protein than women. However, women had a significantly higher intake of fruit and vegetables (Figure 2B).

Daily mood and lifestyle ratings differed insofar that women reported higher levels of anxiety but also rated their social interactions of a higher quality. Trait anxiety levels were also higher in women than in men. No other significant differences between men and women were found.

### Weekly averages of daily data

We investigated whether wellbeing, anxiety and excitement was associated with averages of the diary data in inter-individual models. Based on the multiple regression models, and as shown in Table 2, we found that fruit and vegetable intake correlated with wellbeing (B = 0.01, CI = 0.00–0.02, *p* = 0.013) alongside sleep (B = 0.05, CI = 0.03–0.07, *p* < 0.001), social interaction quality (B = 0.10, CI = 0.06–0.13, *p* < 0.001) and male gender (B = 0.86, CI = 0.01–1.71, *p* = 0.048). Anxiety was significantly associated with social interaction quality (B = –0.01, CI = –0.02 to –0.00, *p* = 0.007) and male gender (B = –0.45, CI = –0.72 to –0.19, *p* = 0.001). Finally, excitement correlated with sleep quality (B = 0.01, CI = 0.00–0.02, *p* = 0.012).

### Mediation analyses

**FIGURE 3:**
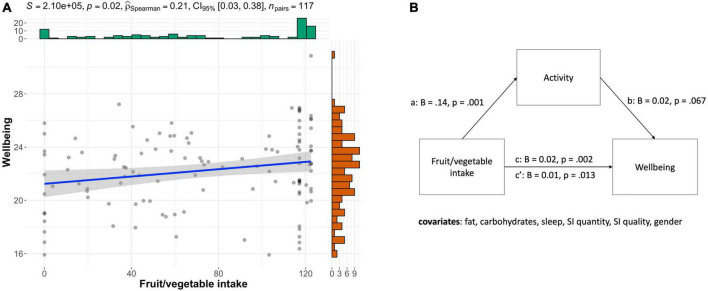
Fruit and vegetable intake affects wellbeing which is partially mediated by activity. **(A)** Scatterplot showing that fruit and vegetable intake correlates positively with wellbeing (rho = 0.21, *p* = 0.021). **(B)** Mediation model, illustrates that higher levels of fruit and vegetable intake were associated with more activity on average (*a*) and a higher level of wellbeing (*c*). Activity showed a non-significant positive trend for wellbeing (*b*). After accounting for the indirect effect, the direct effect remained significant, meaning fruit/veg intake contributes to wellbeing independently of activity (c’).

Next, we tested if concurrent lifestyle (activity, sleep, social interactions) mediated the effect of food intake on wellbeing while controlling for all other lifestyle factors. To validate using a mediation model, we first tested if fruit and vegetable, fat and carbohydrate intake each regress onto wellbeing, which revealed that only fruit and vegetable intake significantly correlated with wellbeing (*B* = 0.02, SE = 0, *t* = 3.20, *p* = 0.002). Next, we tested whether the independent variable fruit and vegetable intake regressed onto the mediators (activity, sleep, social interactions). Fruit and vegetable intake correlated with activity (*B* = 0.14, SE = 0.04, *t* = 3.35, *p* = 0.001) but neither sleep (*B* = –0.02, SE = 0.041, *t* = –0.51, *p* = 0.614) nor quality of social interaction (*B* = –0.00, SE = 0.03, *t* = –0.05, *p* = 0.960). Thus, we ran a mediation model to test whether activity mediated the effect of fruit and vegetable intake on wellbeing (Figure 3). Indeed, this model revealed that the difference in activity partially mediated the direct effect of fruit and vegetable intake on wellbeing (c’, *B* = 0.01, SE = 0.01, *t* = 2.52, *p* = 0.013) compared to the total effect (c, *B* = 0.02, SE = 0, *t* = 3.20, *p* = 0.002; bootstrapped indirect effect (B = 0.03, SE = 0, 95% CI –0.00–0.01).

### Daily and lagged analyses

We also tested intra-individual associations between daily fruit and vegetable, fat and carbohydrate intake and changes in wellbeing using linear mixed-effects models controlling for wellbeing, anxiety, or excitement of the same day, respectively. The results for same-day analyses are shown in Table 3. Same-day wellbeing correlated with fruit and vegetable intake while controlling for same-day sleep, activity and quality, and quantity of social interactions and the previous day’s wellbeing. Neither anxiety nor excitement were associated with diet, but by same-day lifestyle factors.

**TABLE 3:** Same-day associations between diet and lifestyle factors and measures of wellbeing and mood, using linear mixed-effects models.

DV	IV	Coefficient	95% CI	*P*
Wellbeing	Intercept	9.16	5.74–12.58	**<0.001**
	Fruit and vegetable	0.01	0.00–0.01	**0.002**
	Fat	0.01	–0.02–0.04	0.531
	Carbohydrates	0.01	–0.01–0.02	0.552
	Sleep	0.03	0.02–0.04	**<0.001**
	Activity	0.02	0.01–0.03	**<0.001**
	SI^a^ quality	0.07	0.06–0.08	**<0.001**
	SI^a^ quantity	0.01	0.00–0.03	**0.018**
	Previous day wellbeing	0.15	0.09–0.22	**<0.001**
	Gender (male)	0.56	–0.16–1.28	0.129
	Random effects			
	N_id_	109		
	Observations	475		
	Marginal R^2^/Cond. R^2^	0.462/0.588		
Anxiety	Intercept	2.11	1.03–3.19	**<0.001**
	Fruit and vegetable	–0.00	–0.00–0.00	0.686
	Fat	0.00	–0.01–0.01	0.611
	Carbohydrates	0.01	–0.00–0.01	0.075
	Sleep	–0.01	–0.01 to -0.00	**0.001**
	Activity	–0.00	–0.00–0.00	0.445
	SI^a^ quality	–0.01	–0.01 to –0.00	**0.001**
	SI^a^ quantity	–0.00	–0.01 to –0.00	**0.044**
	Previous day anxiety	0.12	0.04–0.20	**0.004**
	Gender (male)	–0.29	–0.52 to -0.05	**0.019**
	Random effects			
	N_id_	109		
	Observations	479		
	Marginal R^2^/Cond. R^2^	0.144/0.336		
Excitement	Intercept	1.21	0.14–2.27	**0.027**
	Fruit and vegetable	0.00	–0.00–0.00	0.964
	Fat	0.00	–0.01–0.01	0.756
	Carbohydrates	–0.00	–0.01–0.00	0.560
	Sleep	0.01	0.00–0.01	**0.001**
	Activity	0.01	0.00–0.01	**0.001**
	SI^a^ quality	0.01	0.01–0.02	**<0.001**
	SI^a^ quantity	0.00	–0.00–0.01	0.119
	Previous day excitement	0.08	–0.00–0.16	0.055
	Gender (female)	0.18	–0.05–0.41	0.129
	Random effects			
	N_id_	109		
	Observations	477		
	Marginal R^2^/Cond. R^2^	0.241/0.407		

^a^Social interaction. All independent variables were entered simultaneously. The bold values mean *p* < 0.05.

We also tested 1-day (Supplementary Table 1) and 2-day-lagged (Supplementary Table 2) associations of fruit and vegetable, fat and carbohydrate intake on wellbeing, anxiety, and excitement each controlling for same-day lifestyle factors revealing similar patterns. For 1-day lags none of the dietary components correlated with wellbeing, anxiety or excitement (all *p* > 0.296). Instead, daily wellbeing was significantly associated with lifestyle factors sleep, activity, social interaction quality, and the previous day’s level of wellbeing (all *p* = 0.001 or < 0.001). Anxiety was correlated with sleep and quality of social interactions (all *p* < 0.001), the previous day’s level of anxiety (*p* = 0.002) as well as male gender (*p* = 0.029). Finally, excitement was associated with sleep, activity, social interaction quality (all *p* = 0.001 or < 0.001), and the previous day’s level of excitement (*p* = 0.018). Two-day lagged associations did not reveal any significant diet associations when accounting for lifestyle factors in the same model (all *p* > 0.184).

### Exploratory analyses

We explored correlations between mental health questionnaires and individuals’ average dietary and lifestyle behaviors. In Table 4 we report Pearson correlations between baseline mental health and wellbeing questionnaires (as dependent variables) and diet and lifestyle variables. We found that fat intake correlates positively with trait anxiety (*r* = 0.30, *p* = 0.007). In addition, self-rated sleep quality and social interaction quality significantly correlate with all dependent variables.

**TABLE 4:** Pearson correlations between baseline mental health and wellbeing questionnaires and diet and lifestyle outcomes.

	Wellbeing	Anxiety	Depressive symptoms	Perceived stress
Fruit and vegetable	0.21	–0.10	–0.16	–0.12
Fat	–0.19	0.30**	0.13	0.22
Carbohydrates	0.20	–0.24	–0.18	–0.19
Sleep	0.39***	–0.34**	–0.36***	–0.37***
Activity	0.23	–0.20	–0.27*	–0.21
Social interaction quality	0.43***	–0.32**	–0.39***	–0.29*
Social interaction quantity	0.19	–0.03	–0.17	–0.06

P-value adjustment method: Bonferroni; *p < 0.05; **p < 0.01; ***p < 0.001. Significance levels were corrected for multiple comparisons for each DV separately. The bold values mean *p* < 0.05.

### Association with the dietary inflammatory index

**FIGURE 4:**
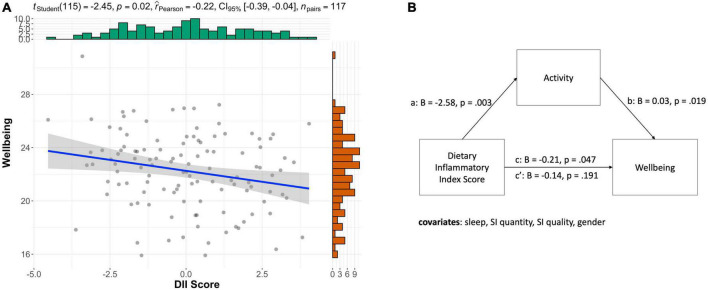
Dietary inflammatory index, wellbeing, and activity. **(A)** Negative correlation between DII and average wellbeing (*r* = 0.22, *p* = 0.016). **(B)** Mediation model illustrating that a more inflammatory diet was associated with being less active (*a*) and reporting lower levels of wellbeing (*c*). While more activity was also associated with higher wellbeing (*b*).

As inflammation is a possible mechanism by which diet affects mental wellbeing, we tested if a high Dietary Inflammatory Index (DII) is associated with lower wellbeing and higher levels of anxiety. DII score correlated significantly with averaged daily wellbeing (*r* = –0.20, *p* = 0.027, Figure 4A) but not with anxiety (*r* = 0.17, *p* = 0.063) or excitement (*r* = –0.09, *p* = 0.332).

Based on the mediation effect we found above, we also tested if average daily lifestyle (i.e., activity, sleep, social interactions) mediated the effect of an inflammatory diet on wellbeing. DII negatively correlated with wellbeing (*B* = –0.20, SE = 0.10, *t* = –2.00, *p* = 0.047). As for possible mediators, DII negatively correlated with activity (*B* = –2.58, SE = 0.86, *t* = –3.00, *p* = 0.003) but neither sleep (B = –0.37, SE = 0.84, *t* = –0.44, *p* = 0.658) nor social interaction quality (*B* = –0.15, SE = 0.62, *t* = –0.23, *p* = 0.815). Therefore, we tested for a mediation of activity only. We found that activity fully mediated the direct effect (c’) of the dietary inflammatory score on wellbeing (*B* = –0.14, SE = 0.11, *t* = –1.32, *p* = 0.191) compared to the total effect (c, *B* = –0.21, SE = 0.10, *t* = –2.00, *p* = 0.047; bootstrapped indirect effect (*B* = –0.07, SE = 0.04, 95% CI –0.15 to 0.00) as shown in Figure 4B.

### Gender-specific effects

**FIGURE 5:**
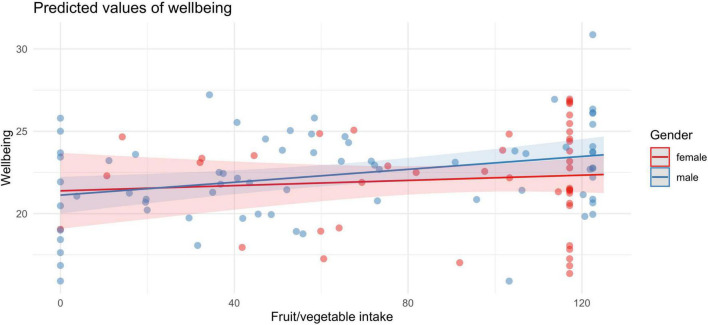
Both men (blue) and women (red) showed a positive association between fruit and vegetable intake and wellbeing.

Given that female participants consumed significantly more fruits and vegetables compared to men [M_female_ = 91.96 (35.17), M_male_ = 62.48 (44.89), *p* = 0.009], we explored if the strength of the association between fruit and vegetable intake and wellbeing differed depending on gender. However, as shown in Figure 5, an estimation of the marginal means of linear trends did not show that the interaction between gender and fruit/vegetable intake was significantly different (*B* = –0.01, *p* = 0.409).

Given that age and BMI significantly differed between male and female participants (see Table 1), we wondered if these variables could account for the gender effects we found. While fruit and vegetable intake correlated negatively with BMI (*r* = –0.18, *p* = 0.048), wellbeing did not (*r* = –0.07, *p* = 0.440). However, age did not correlate with either wellbeing (*r* = 0.05, *p* = 0.575) or fruit and vegetable intake (*r* = 0.13, *p* = 0.166).

The effect of Dietary Inflammatory Index on wellbeing was also independent of gender (*B* = –0.10, *p* = 0.732). Furthermore, we were curious as to whether gender differently interacted with social interaction quality and wellbeing. This was not the case (*B* = 0.05, *p* = 0.126); for both genders, social interaction quality positively affected wellbeing (for women: *B* = 0.15, *p* < 0.001; for men: *B* = 0.10, *p* < 0.001). Likewise, sleep was positively associated with wellbeing in both genders (overall contrast: *B* = 0.07, *p* = 0.007, for women: *B* = 0.12, *p* < 0.001; for men: *B* = 0.05, *p* = 0.003).

## Discussion

This preregistered study investigated how dietary intake affected mood and wellbeing alongside lifestyle factors during COVID-19-lockdown. Previous studies showed that dietary components (7, 13, 14), exercise and sleep impacted on mental health and wellbeing (4). We were also interested in social interaction as a contributor to wellbeing (29), since social distancing measures were so prominent during lockdowns.

We hypothesized that food intake was associated with (1) wellbeing; (2) anxiety; and (3) excitement and tested between- and within-person relationships while controlling for concurrent lifestyle factors. Both in our regression models, as well as mediation analysis, we observed that fruit and vegetable intake correlated with wellbeing, while this was partially mediated by physical activity.

### Diet and lifestyle in the context of COVID-19-lockdown

The pandemic context brought about changes in diet, sleep, and activity (52), which brought about increased negative mood (52–55) and lower wellbeing (1). Lower dietary quality was associated with poor mood and may have been used to regulate emotions (55). The present findings complement this by providing evidence that inversely, consuming healthier foods, i.e., fruit and vegetable, were linked with more wellbeing. Work by Cecchetto and colleagues’ investigated whether social factors (amongst others) contributed to dysfunctional eating habits during the pandemic (36). However, a more holistic approach of lifestyle factors that include physical activity, sleep, dietary intake and social interaction to investigate their joint effect on wellbeing, anxiety and excitement had thus far been lacking.

Undergoing lockdown may have undermined the impact of diet on mood when accounting for other healthful behaviors. For example, mood affects the likelihood of making healthy food choices mediated by physical activity (56). The authors suggest that people engage in healthy *lifestyles* rather than isolated health behaviors, i.e., being physically active goes together with making healthier dietary choices (56). Our data support this notion; high intakes of fruit and vegetable as well as physical activity were associated with increased levels of wellbeing.

Additionally, other lifestyle factors may have gained importance during this period. Highly active people experienced significant declines in quality of sleep and wellbeing during lockdown as compared to sedentary individuals (2). Furthermore, dramatic declines in physical activity, especially walking, were recorded due to lockdown restrictions and increased home-office hours or job termination in this period (57). Being active outdoors compared to indoors may contribute further to mental wellbeing in addition to the exercise itself (58). The more time spent outdoors in daylight lowered the risk of depression, low mood and added to happiness (59). Thus, lockdown restrictions may have magnified beneficial effects of physical activity during lockdown, and even more so when activity happened outdoors.

Finally, social interactions were greatly affected by social distancing measures. For example, social media use increased during the pandemic (60) and was linked to poor mental health in a large cross-country sample (61), and increased the odds of experiencing anxiety in a Chinese (62) and American sample (60). While greater social connectedness was associated with less perceived stress during the pandemic (63). In line with the existing literature, we found that the quality but not quantity of social interactions correlated with mood and wellbeing in almost all analyses, echoing previous findings (64). To our knowledge, social interactions have not yet been considered in models alongside diet, sleep, and activity. Our findings suggest that during lockdown the quality of social interactions plays a key role when examining the relationship between diet, wellbeing, and mood.

### Evaluating dietary intake

Dietary intake can be analyzed in many different ways. Here we focused on specific dietary components. Fat, carbohydrates, and fruit and vegetable intake had been identified in the literature to play a key role in mood and wellbeing (13, 14, 65). Our findings supported the role of fruit and vegetable intake in concurrent wellbeing. Furthermore, we found an association between trait anxiety and fat intake, whereby higher fat intake correlated with greater state anxiety. However, we did not find that total fat intake correlated with daily anxiety levels when controlling for other lifestyle factors.

Additionally, we calculated the dietary inflammatory index—a well-established measure of a diet’s inflammatory potential (16). We found that DII score correlated negatively with average wellbeing but not with anxiety or excitement. DII score has been found to correlate with wellbeing before (66). We also found that the effect of DII on wellbeing was fully mediated by activity.

We examined whether dietary intake was associated with wellbeing, anxiety, and excitement. However, vice versa, it is an interesting question whether negative mood and mental health issues can drive low-quality food intake. Neither longitudinal (67) nor short-term evidence, 1- or 2-day lagged associations (13, 14) support this idea. However, a recent study conducted during COVID-19-lockdown found that mood states were linked to the intake of fruit, vegetables, and fish, which were partially mediated by physical exercise load (56). The authors suggested that some participants may have actively changed their exercise and food intake behavior to deal with the anticipated challenges on mental health during lockdown (56). Importantly, these authors included exercise as a lifestyle factor to investigate the relationship between mood and diet. In sum, the differences between studies may be due to the unusual circumstances of the pandemic as well as the mediating factor of physical exercise, which was affected by pandemic restrictions (27, 57). Finally, Amatori et al. did not report testing the reverse direction, i.e., whether dietary intake was correlated with mood states (56).

### Gender-specific effects

Here we found gender differences in food intake, anxiety levels, and quality of social interaction. In particular, women consumed more fruit and vegetables but fewer calories from protein than men. This is in line with previous work demonstrating gender differences in dietary intake (68–70). For instance, women across 23 countries showed greater beliefs in the importance of healthy eating as evident by higher intake of fruit and fiber-rich foods (70). In this study, women reported higher baseline and concurrent anxiety levels than men in this study, consistent with previous findings (71). But we did not find that higher fruit and vegetable intake was associated with lower anxiety ratings, contrary to what has been reported elsewhere (8). Eating more fruit and vegetables also did not affect wellbeing to a greater extent than men. It is currently unclear why women’s mood did not benefit from fruit and vegetable intake more so than men despite higher intake, or why anxiety levels were unaffected by higher fruit and vegetable intake. Thus, more research is needed to better understand mechanistic links between diet, body, brain, and gender interactions.

### Strengths and limitations

A few limitations need to be considered. First, due to the acute nature of the pandemic, we lack a baseline dietary assessment, and cannot make claims whether dietary intake has changed in response to the lockdown. Second, as with any self-report study, these measures underlie self-reporting biases. For example, self-reported caloric intake is likely underreported. Underreporting is a common problem in self-reported dietary data (72). Note that we also chose to exclude individuals with mental health diagnoses and severe depressive symptoms, therefore our findings cannot be generalized to subclinical and clinical populations.

Strengths of this study include the use of preregistration of hypotheses and analyses before data collection. Considering that dietary intake alongside multiple lifestyle factors and social aspects is still understudied, highlights the need for a holistic approach to assess lifestyle with mood and mental health outcomes. Furthermore, we were able to collect a rich data set by assessing baseline parameters of mental health and lifestyle followed by a 7-day diary of food intake. Using such a food diary, rather than a 24-hr recall, alongside concurrent mood and lifestyle factors allowed us to explore both inter- and intra-individual fluctuations of these variables. The findings of this study are limited to a relatively young German population, and further research would be needed to determine if the same effects can be found for different age groups and specific health groups. An interesting avenue for future studies would be to investigate whether the dynamic between mood, diet, lifestyle, and social interactions still holds beyond the acute lockdown situation observed in this study, and whether this extends to different individuals such as clinical populations.

## Conclusion

Our results showed that, on average, fruit and vegetable intake contributed to wellbeing alongside sleep and social interaction quality. Examining day-to-day associations showed that fruit and vegetable intake on the same day promoted wellbeing, while this was not the case for the next day or second day time lags. Instead, sleep, activity, and social interactions were associated with wellbeing in the context of lockdown during the COVID-19 pandemic. Importantly, associations between fruit and vegetable intake were partially mediated by physical activity. These findings highlight the need for an integrated way of assessing lifestyle factors and gender in future studies. As pandemics are thought to appear more frequently due to diminishing biodiversity (73), strategies to protect mental health and wellbeing become more important than ever, especially because access to mental health care remains limited for many. Therefore, reducing the risk for adverse psychological effects *via* lifestyle behaviors such as diet, activity, and sleep remains a promising strategy [for a meta-review on lifestyle psychiatry see Firth et al. (4)].

In conclusion, a combination of physical activity, good sleep, and daily high-quality social interactions as well as a diet rich in fruit and vegetables and a low inflammatory potential (i.e., diets high in minerals and vitamins, such as fruit and vegetables, but low in saturated fats) appears to promote better mood and wellbeing in stressful circumstances such as a lockdown during a global pandemic. Our research result offers a novel perspective of dietary and lifestyle recommendations that can be provided in times of high uncertainty, such as pandemic situation.

## Data availability statement

The raw data supporting the conclusions of this article will be made available by the authors, without undue reservation.

## Ethics statement

The studies involving human participants were reviewed and approved by the Humboldt University of Berlin. The patients/participants provided their written informed consent to participate in this study.

## Author contributions

A-KM and SP: conceptualization and project administration. A-KM: investigation, visualization, and writing—original draft preparation. A-KM, AL, and SP: methodology and formal analysis. A-KM, AL, DT, and SP: writing—review and editing. AL and SP: supervision. SP: funding acquisition. All authors have read and agreed to the published version of the manuscript.

## Footnotes

1
https://osf.io/nqhjf

2
https://www.bundesregierung.de/breg-de/themen/coronavirus/corona-massnahmen-1734724
