# Supplementary material for: The impact of diet and lifestyle on wellbeing in adults during COVID-19 lockdown
Source: Front Nutr. 2022 Oct 6;9:993180. doi: 10.3389/fnut.2022.993180 (PMC9582278; doi:10.3389/fnut.2022.993180)
Supplement: Supplementary file 1 [file Table_1.docx]

Supplementary Table 1. One-day lagged effects of diet and lifestyle factors on measures of wellbeing and mood, using linear mixed-effects models. All independent variables were entered simultaneously.

| **DV** | **IV** | **Coefficient** | **95% CI** | **p** |
| --- | --- | --- | --- | --- |
| Wellbeing | Intercept | 13.13 | 9.86 – 16.40 | **<0.001** |
|  | Fruit and vegetable | -0.00 | -0.01 – 0.00 | 0.296 |
|  | Fat | -0.01 | -0.04 – 0.02 | 0.533 |
|  | Carbohydrates | -0.01 | -0.02 – 0.01 | 0.516 |
|  | Sleep | 0.03 | 0.02 – 0.04 | **<0.001** |
|  | Activity | 0.03 | 0.02 – 0.04 | **<0.001** |
|  | SI^1^ quality | 0.06 | 0.05 – 0.08 | **<0.001** |
|  | SI^1^ quantity | 0.01 | -0.00 – 0.02 | 0.052 |
|  | Previous day wellbeing | 0.12 | 0.05 – 0.18 | **0.001** |
|  | Gender (male) | 0.30 | -0.52 – 1.11 | 0.474 |
|  | Random Effects |  |  |  |
|  | N_id_ | 113 |  |  |
|  | Observations | 475 |  |  |
|  | Marginal R^2^/ Cond. R^2^ | 0.401/0.605 |  |  |
| Anxiety | Intercept | 3.26 | 2.24 – 4.29 | **<0.001** |
|  | Fruit and vegetable | -0.00 | -0.00 – 0.00 | 0.543 |
|  | Fat | -0.00 | -0.01 – 0.01 | 0.595 |
|  | Carbohydrates | -0.00 | -0.01 – 0.00 | 0.450 |
|  | Sleep | -0.01 | -0.01 – -0.00 | **<0.001** |
|  | Activity | -0.00 | -0.00 – 0.00 | 0.355 |
|  | SI^1^ quality | -0.01 | -0.01 – -0.00 | **<0.001** |
|  | SI^1^ quantity | -0.00 | -0.00 – 0.00 | 0.509 |
|  | Previous day anxiety | 0.13 | 0.05 – 0.21 | **0.002** |
|  | Gender (male) | -0.26 | -0.48 – -0.03 | **0.029** |
|  | Random effects |  |  |  |
|  | N_id_ | 113 |  |  |
|  | Observations | 479 |  |  |
|  | Marginal R^2^/ Cond. R^2^ | 0.140/0.310 |  |  |
| Excitement | Intercept | 1.46 | 0.45 – 2.48 | **0.005** |
|  | Fruit and vegetable | -0.00 | -0.00 – 0.00 | 0.472 |
|  | Fat | -0.00 | -0.01 – 0.01 | 0.562 |
|  | Carbohydrates | -0.00 | -0.01 – 0.00 | 0.504 |
|  | Sleep | 0.01 | 0.00 – 0.01 | **0.001** |
|  | Activity | 0.01 | 0.00 – 0.01 | **<0.001** |
|  | SI^1^ quality | 0.01 | 0.01 – 0.02 | **<0.001** |
|  | SI^1^ quantity | 0.00 | -0.00 – 0.01 | 0.206 |
|  | Previous day excitement | 0.09 | 0.02 – 0.17 | **0.018** |
|  | Gender (male) | 0.11 | -0.12 – 0.33 | 0.352 |
|  | Random effects |  |  |  |
|  | N_id_ | 113 |  |  |
|  | Observations | 477 |  |  |
|  | Marginal R^2^/ Cond. R^2^ | 0.233/0.365 |  |  |

^1^Social interaction
