# Supplementary material for: The impact of diet and lifestyle on wellbeing in adults during COVID-19 lockdown
Source: Front Nutr. 2022 Oct 6;9:993180. doi: 10.3389/fnut.2022.993180 (PMC9582278; doi:10.3389/fnut.2022.993180)
Supplement: Supplementary file 2 [file Table_2.docx]

Supplementary Table 2. Two-day lagged associations between diet and lifestyle factors and measures of wellbeing and mood, using linear mixed-effects models. All independent variables were entered simultaneously.

| **DV** | **IV** | **Coefficient** | **95% CI** | **p** |
| --- | --- | --- | --- | --- |
| Wellbeing | Intercept | 11.68 | 8.45 – 14.90 | **<0.001** |
|  | Fruit and vegetable | -0.00 | -0.01 – 0.00 | 0.234 |
|  | Fat | -0.01 | -0.04 – 0.02 | 0.577 |
|  | Carbohydrates | -0.00 | -0.02 – 0.01 | 0.747 |
|  | Sleep | 0.02 | 0.01 – 0.03 | **<0.001** |
|  | Activity | 0.02 | 0.01 – 0.03 | **<0.001** |
|  | SI^1^ quality | 0.07 | 0.06 – 0.08 | **<0.001** |
|  | SI^1^ quantity | 0.02 | 0.01 – 0.03 | **0.003** |
|  | Previous day wellbeing | 0.14 | 0.08 – 0.21 | **<0.001** |
|  | Gender (male) | 0.55 | -0.22 – 1.33 | 0.160 |
|  | Random Effects |  |  |  |
|  | N_id_ | 113 |  |  |
|  | Observations | 464 |  |  |
|  | Marginal R^2^/ Cond. R^2^ | 0.442/0.600 |  |  |
| Anxiety | Intercept | 2.38 | 1.38 – 3.39 | **<0.001** |
|  | Fruit and vegetable | 0.00 | -0.00 – 0.00 | 0.184 |
|  | Fat | 0.00 | -0.01 – 0.01 | 0.424 |
|  | Carbohydrates | 0.00 | -0.00 – 0.01 | 0.455 |
|  | Sleep | -0.00 | -0.01 – -0.00 | **0.014** |
|  | Activity | -0.00 | -0.00 – 0.00 | 0.467 |
|  | SI^1^ quality | -0.01 | -0.01 – -0.00 | **<0.001** |
|  | SI^1^ quantity | -0.00 | -0.01 – 0.00 | 0.129 |
|  | Previous day anxiety | 0.15 | 0.07 – 0.24 | **<0.001** |
|  | Gender (male) | -0.30 | -0.52 – -0.08 | **0.007** |
|  | Random effects |  |  |  |
|  | N_id_ | 113 |  |  |
|  | Observations | 468 |  |  |
|  | Marginal R^2^/ Cond. R^2^ | 0.151/0.289 |  |  |
| Excitement | Intercept | 0.90 | -0.10 – 1.90 | 0.078 |
|  | Fruit and vegetable | -0.00 | -0.00 – 0.00 | 0.663 |
|  | Fat | 0.00 | -0.01 – 0.01 | 0.436 |
|  | Carbohydrates | 0.00 | -0.01 – 0.01 | 0.911 |
|  | Sleep | 0.00 | 0.00 – 0.01 | **0.027** |
|  | Activity | 0.01 | 0.00 – 0.01 | **<0.001** |
|  | SI^1^ quality | 0.01 | 0.01 – 0.02 | **<0.001** |
|  | SI^1^ quantity | 0.00 | -0.00 – 0.01 | 0.071 |
|  | Previous day excitement | 0.10 | 0.02 – 0.19 | **0.014** |
|  | Gender (male) | 0.21 | -0.02 – 0.44 | 0.077 |
|  | Random effects |  |  |  |
|  | N_id_ | 113 |  |  |
|  | Observations | 466 |  |  |
|  | Marginal R^2^/ Cond. R^2^ | 0.234/0.400 |  |  |

^1^Social interaction
